# Soccer results affect subjective well-being, but only briefly: a smartphone study during the 2014 FIFA World Cup

**DOI:** 10.3389/fpsyg.2015.00497

**Published:** 2015-05-12

**Authors:** Stefan Stieger, Friedrich M. Götz, Fabienne Gehrig

**Affiliations:** Department of Psychology, University of KonstanzKonstanz, Germany

**Keywords:** subjective well-being, soccer world cup, science app, smartphone study, positive psychology, emotional contagion

## Abstract

The current research examined the effects of soccer match results on spectators’ subjective well-being. Across the group stage of the soccer World Cup 2014, German-speaking participants indicated their well-being three times per day through a smartphone-based science app. In line with proposed hypotheses, comparisons of data taken after the three matches of the German national team showed robust effects, revealing that well-being was higher among spectators than non-spectators, with effects increasing as a function of goal difference. Moreover, this gain in well-being was only found in spectators supporting the German soccer team, allowing us to rule out a general emotional contagion effect affecting all spectators. Although soccer results are associated with national identity and pride, their effects on subjective well-being were short-lived and only affected supporters.

## Introduction

International sporting events have an influence on our emotions: they are sources of joy and frustration, anger and pride, depression and enthusiasm. For instance, the German soccer national team’s unexpected success at the 2006 soccer World Cup led to increased identification with the team and greater national pride among soccer fans and as a result higher self-worth. This phenomenon has been referred to as the “feel-good effect at mega sports events“ (for a review, see Maennig and Porsche, 2008).

This effect is consistent with the key assumptions of the Team Identification-Social Psychological Health Model, which proposes that identification with a sports team facilitates social connections that have an impact on social psychological health (Wann, 2006). According to Pawlowski et al. (2014), hosting and consequently being able to attend such events is associated with higher subjective well-being. More to the point, it appears that attendance at such events principally elicits increased pride, which in turn results in an enhancement in subjective well-being. Even so, pride is traditionally conceived of as a stable characteristic, but recent research suggests instead that pride has several facets with stable and unstable characteristics (e.g., authentic vs. hubristic pride; Tracy and Robins, 2007). Given that a national team’s performance at major tournaments, such as the World Cup, has been shown to alter well-being, it might be argued that such a link does indeed exist, regardless of its specific underpinnings (Elling et al., 2014).

In general, subjective well-being, one of the key concepts of positive psychology, is closely tied to happiness and should be treated as an important parameter to indicate the success of a society as a whole (Diener, 2000). While being broadly defined as the product of frequent positive affect, infrequent negative affect, and global life satisfaction (Myers and Diener, 1995), subjective well-being is thought to reflect both stable trait and changeable state elements. Subjective well-being has been demonstrated to vary according to numerous factors, both at the national level (i.e., income, relative equality, degree of individualism, social welfare, political stability, democracy, life expectancy, mental disorders: Jorm and Ryan, 2014; and human rights: Diener et al., 1995), as well as at the individual level (i.e., neuroticism, extraversion, and openness to experience: Headey and Wearing, 1989).

In spite of these stable determinants of subjective well-being, researchers have stressed that it is important to consider short-term experiences of subjective well-being (DeNeve and Cooper, 1998). In an attempt to devise a consistent theoretical framework, Headey and Wearing (1989) put forward a dynamic equilibrium model, postulating that changes in subjective well-being reflect deviations from an individual’s ordinary equilibrium level, shaped by stable characteristics that occur following either unusually favorable or unusually adverse events. Drawing from this, we tested whether the results of the German soccer national team during the group stage of the World Cup 2014 in Brazil would serve as a possible example of events inducing such deviations, which in turn would affect spectators’ well-being.

Overall, the effects on well-being are most pronounced for failures. For instance, psychological distress among English Premier League soccer fans rose considerably after their favorite team had been relegated (Banyard and Shevlin, 2001). Such distress may even evoke severe impairments, including circulatory diseases, such as acute myocardial infarction and stroke when the supported team experiences a loss (Kirkup and Merrick, 2003). Furthermore, stressful soccer matches have been found to double the risk of an acute cardiovascular event (Wilbert-Lampen et al., 2008). Analogously, sporting teams’ wins in important matches were related to significant declines in suicide rates (Fernquist, 2000).

In summary, it appears that meaningful sporting events in general, and the supported team’s results in particular, have a bearing on different aspects of subjective well-being, such as health and general mood. However, the robustness of these conclusions is limited for two reasons: First, the variables under investigation have often been measured after the event has taken place, i.e., participants had to *remember* their subjective well-being. Second, past studies have often relied on active high-committed sport fans (e.g., soccer fans in a stadium), i.e., whether an effect is transferable to the general population is questionable.

In the present study, we sought to change this state of affairs by applying an Experience Sampling Methodology (ESM; real-time and multiple time point measurements in the field) using a smartphone app that was especially designed for this purpose. We investigated whether and the extent to which the results of the German soccer team during the group stage of the World Cup 2014 affected spectators’ subjective well-being directly after the matches. Doing so allowed us to shed new light on these afore-mentioned rather ill-understood and rarely researched dynamics. Furthermore, we are not aware of any existing studies that have monitored the immediate effects of the results of one’s sports team on subjective well-being.

Concretely, we hypothesized that the results of the matches in which the German soccer team played would affect spectators’ well-being if they were interested in the matches and supported the German team in general. Consequently, it was expected that successes would be associated with increased spectators’ well-being, whereas a reversed pattern should emerge in case of defeat. We had no specific expectations about the duration of these effects.

Moreover, we were interested in the nature of a possible effect. It has often been suggested that competitions of national implications affect national identity and pride (e.g., Elling et al., 2014). If we were to find increased subjective well-being after a win, could this be attributed to a general phenomenon (e.g., emotional contagion) affecting all spectators irrespective of their support for the German soccer team, or would we be dealing with a rather specific phenomenon (e.g., only affecting spectators that are soccer fans supporting their team)? Therefore, we formulated a second hypothesis stating that a possible effect on subjective well-being should be grounded in a general mechanism (e.g., emotional contagion) affecting all spectators and not just those supporting the team.

## Materials and Methods

### Participants

The sample consisted of 213 participants (39% men, 58% women, 3% did not disclose their sex), recruited by word-of-mouth, constituting a community-based sample. Participant age ranged from 16 to 59 years (*M* = 24.5, *SD* = 8.4). Participants were predominantly of German nationality (93.4%; Switzerland, 1.4%, other countries, 1.0%, 4.2% did not disclose their nationality).

### Measures

Subjective well-being was measured by the single-item “how is your current well-being?” that had to be answered on a visual slider scale (VSS) ranging from 0 to 100 (for a similar procedure, see MacKerron and Mourato, 2013). The VSS was chosen as it has been shown to yield better data quality than traditional alternatives in internet-based research (e.g., radio button scales; for a similar application, see Reips and Funke, 2008). Moreover, it has turned out to be particularly useful when implemented in single-item measures to assess key variables of positive psychology (e.g., quality of life; de Boer et al., 2004).

A post-test questionnaire was designed to assess behaviors regarding the World Cup. Thus, four dichotomous items were presented, asking (1) whether the respondent had followed the World Cup, (2) whether s/he had watched the team’s first match against Portugal, (3) whether s/he had watched the team’s second match against Ghana, (4) and lastly, whether s/he had watched the team’s final group stage match against the US.

Furthermore, we asked respondents about the intensity of support, i.e., how strongly they supported the German national team in general at the World Cup using a 5-point scale, anchored at 1 (not at all) and 5 (very much).

### Smartphone App

An exclusive, study-purpose smartphone app called Well-Being Science App was designed for this work. The app was made freely available through the Google App Store, where participants could directly download the app onto their smartphones. We also programmed back-end server software to communicate with the installed apps, i.e., to store the data and to provide participants with personal statistical graphics (e.g., overall well-being score; weekly statistic).

After having installed the app (and prior to the study’s start), participants were asked to provide informed consent and were asked about basic demographics (age, sex, and nationality). In addition, participants had to state their general well-being (“How is your well-being in general?”) using the same VSS as in the prospective part of the study. After that, the main screen appeared asking for the current well-being using a VSS – this screen was used for the prospective part of the study (i.e., the first three screens were only shown once during the first administration). On this screen, participants also had the possibility to request personal statistics in graphical form.

### Procedure

Participants were German-speaking volunteers recruited by word-of-mouth through friends and relatives of several research assistants. We implemented an ESM (real-time and multiple time point measurements). With ESM, participants had to actively respond to questions through the smartphone app, while being in their natural environment. Participants received a reminder sent out via SMS or WhatsApp by the project investigator three times a day for a period of 2 weeks (i.e., the whole group phase of the soccer World Cup). Although this strategy can be burdensome for participants, it captures participants’ everyday life behavior more accurately than retrospective self-report measures (Conner et al., 2009; Kurtz and Lyubomirsky, 2011) and is less invasive than automated sensor-based systems (e.g., Lane et al., 2011).

The timeframe of the study was determined by the group stage of the FIFA World Cup 2014 that took place in Brazil. The time points of data collection during the days were varied randomly throughout the study within three large time frames (morning: 8:00 AM to 9:30 AM; noon: 12:00 PM to 16:00 PM; evening: 19:00 PM to 23:00 PM). The only exceptions were the days when the soccer matches took place. Here, the third time points were chosen, being directly after the end of the match and not at random.

Once the prospective data collection waves had been completed, an internet-based post-test questionnaire was administered, assessing further demographics, accompanied by a short battery of questions related to the World Cup and some other scales, which are not part of this research question (personality structure – Big Five, mind-wandering). Again, participants were asked about their general well-being using the VSS. Twenty-six participants did not fill in the online questionnaire, which resulted in a final sample size of *N* = 187 participants.

Participation was unpaid and completely voluntary. However, participants received course credit, if needed and were invited to take part in a lottery that offered the chance to win an Amazon gift card of 20€. The entire study was run in German. Participants were told that the study was generally about well-being in daily life; hence participants were unaware about the focus of the study, i.e., the World Cup.

### Anonymity of Data

To guarantee anonymity, a 9-digit random participant number was generated after the installation process. This number was the only possible way to connect collected data through different modes (smartphone data, online questionnaire data). Data were stored in different places (directly on the smartphone, on a web-server) and transmitted through different channels (from smartphones to web-servers, from web-servers to web-applications to create graphics, and so forth). To protect these data we used a secure protocol (i.e., https) and additionally encrypted the participant number whenever sent over the Internet.

### Ethics

Participants were from Germany-speaking countries with clear IRB procedures. This study was conducted in accordance with the Declaration of Helsinki and guidelines of the Department of Psychology, University of Konstanz. All participants provided written informed consent prior to their participation. Data collection was anonymous and no harmful procedures were used. Furthermore, they could withdraw at any time during the study without penalty.

## Results

### Preliminary Results

The mean well-being score for all participants and over time points (*n* = 8,382) replicates past research using the same VSS (*M* = 69.3, *SD* = 22.0; MacKerron and Mourato, 2013: *M* = 66.4, *SD* = 21.6, *d* = 0.13; happiness rating). First, we analyzed whether the time of the day, the day of the week, or participants’ sex and age had an effect on well-being. Well-being constantly rose during the day, starting with a low level in the morning and the highest level approaching midnight [*F*(13,7152) = 9.84, *p* < 0.001], although this effect was weak ($\eta_{p}^{2}$ = 0.02; correlation between well-being and time of the day: *r* (7152) = 0.13, *p* < 0.001). Furthermore, we found that the day of the week had an influence, with Monday, Tuesday, and Wednesday having lower values than Thursday, Friday, Saturday, and Sunday. Although significant [*F*(6,7152) = 2.47, *p* = 0.02], this effect was tiny ($\eta_{p}^{2}$ = 0.002) and a Scheffé *post hoc* test found only one homogeneous group (Scheffé: *p* = 0.254)^1^. Participants’ age and sex also revealed only non-significant, tiny effects (age: *r* (181) = 0.11, *p* = 0.138; sex: *t*(179) = 0.14, *p* = 0.890, *d* = 0.02). Therefore, we did not control for these variables in all further analyses.

### Trait vs. State Well-Being

Furthermore, we analyzed one major limitation of past research by using recalled general assessments of well-being (e.g., “How is your general well-being?”), rather than specific near-time assessments right after the event (e.g., “How is your current well-being?”). In the present study, participants were asked about their general well-being at the beginning of the study, as well as the end of the study, using the same VSS scale as with the daily judgments of current well-being. As expected, the general assessments of well-being at the beginning (*M* = 75.8, *SD* = 16.2) and the end of the study (*M* = 74.9, *SD* = 15.1) did not differ significantly [*t*(159) = 0.725, *p* = 0.470, *d* = 0.08], underlining the trait-nature of this assessment. This is also an indicator that the procedure of constantly giving well-being assessments over a period of 14 days (i.e., in sum 42 single state assessments of well-being) did not significantly alter the overall well-being score (i.e., no obvious intervention effect). However, the mean score of all single state assessments of well-being through the app (*M* = 70.3, *SD* = 12.4) was significantly lower and of medium-to-large effect size than both general assessments (*F* = 13.14, *p* < 0.001, $\eta_{p}^{2}$ = 0.076). This underlines the assumption that trait and state well-being are conceptionally different (e.g., Steyer et al., 1992).

### Influence of Soccer Result on Spectators’ Well-Being: Between-Subject View

We followed a between-subject view by comparing those participants that watched a soccer match (Germany vs. Portugal, Ghana, and USA, respectively) to those who did not watch the matches (spectators vs. non-spectators). Because this procedure reflects a quasi-experimental design, we checked for demographic differences between groups. We could neither find age- nor sex-differences between the groups (all *t*s < 0.74, all χ^2^ < 1.28; all *p*s > 0.26), i.e., both groups were comparable regarding their demographic composition. We hypothesized that soccer spectators’ well-being, assessed directly after the match, would be significantly affected by their national team’s performance, as opposed to non-spectators having not seen the match. As can be seen in **Table 1**, spectators had higher well-being scores than non-spectators for the Germany vs. Portugal match (Germany won 4-0) and the Germany vs. USA match (Germany won 1-0) with medium-to-large effect sizes. No effect was found for the Germany vs. Ghana match probably because it ended with a draw (2–2; for details see **Table 1**).

**Table 1:** Between-subject comparisons of well-being directly after the match between spectators of the respective match and those that did not watch the match.

Germany vs.	Did not watch the match (non-spectators)		Watched the match (spectators)		Between-group comparison		ANCOVA with intensity of support for the German soccer team as a covariate	
	*n*	*M (SD)*	*n*	*M (SD)*	*t*	*d*	*F* test	$\eta_{p}^{2}$
Portugal (4:0)	26	68.3 (18.8)	143	81.2 (21.5)	2.88**	0.64	*F* [1,169] = 2.23	0.01
Ghana (2:2)	15	68.7 (23.4)	126	67.2 (24.9)	-0.22	-0.06	*F* [1,141] = 0.20	<0.01
USA (1:0)	24	68.0 (16.1)	139	76.8 (19.4)	2.39*	0.49	*F* [1,163] = 1.73	0.01

*p < 0.05, **p < 0.01.

*d = Cohen *d* (small: 0.2, medium: 0.5, large: 0.8); $\eta_{p}^{2}$ (small: 0.01, medium: 0.06, large: 0.14); see Cohen (1988).*

Next, we analyzed whether the observed effect for the Portugal and USA matches was driven by the outcome of the match itself or by the intensity of support toward the German soccer team. In the first case, non-interested spectators should also be affected by a win (e.g., through emotional contagion). In the second case, only those who intensively support the German soccer team should be affected. We calculated ANCOVAs to control for the intensity of support. If every spectator is affected by a win, then the intensity of support should not have an influence on the observed found effect between spectators and non-spectators, i.e., the well-being differences should basically remain stable for the Portugal and USA match. As can be seen in **Table 1** (last two columns), after controlling for the intensity of support, all effects were non-significant and of tiny effect size, i.e., a win for the German soccer team only had a positive effect on well-being for those spectators who also supported the team.

### Influence of Soccer Result on Spectators’ Well-Being: Within-Subject View

**FIGURE 1:**
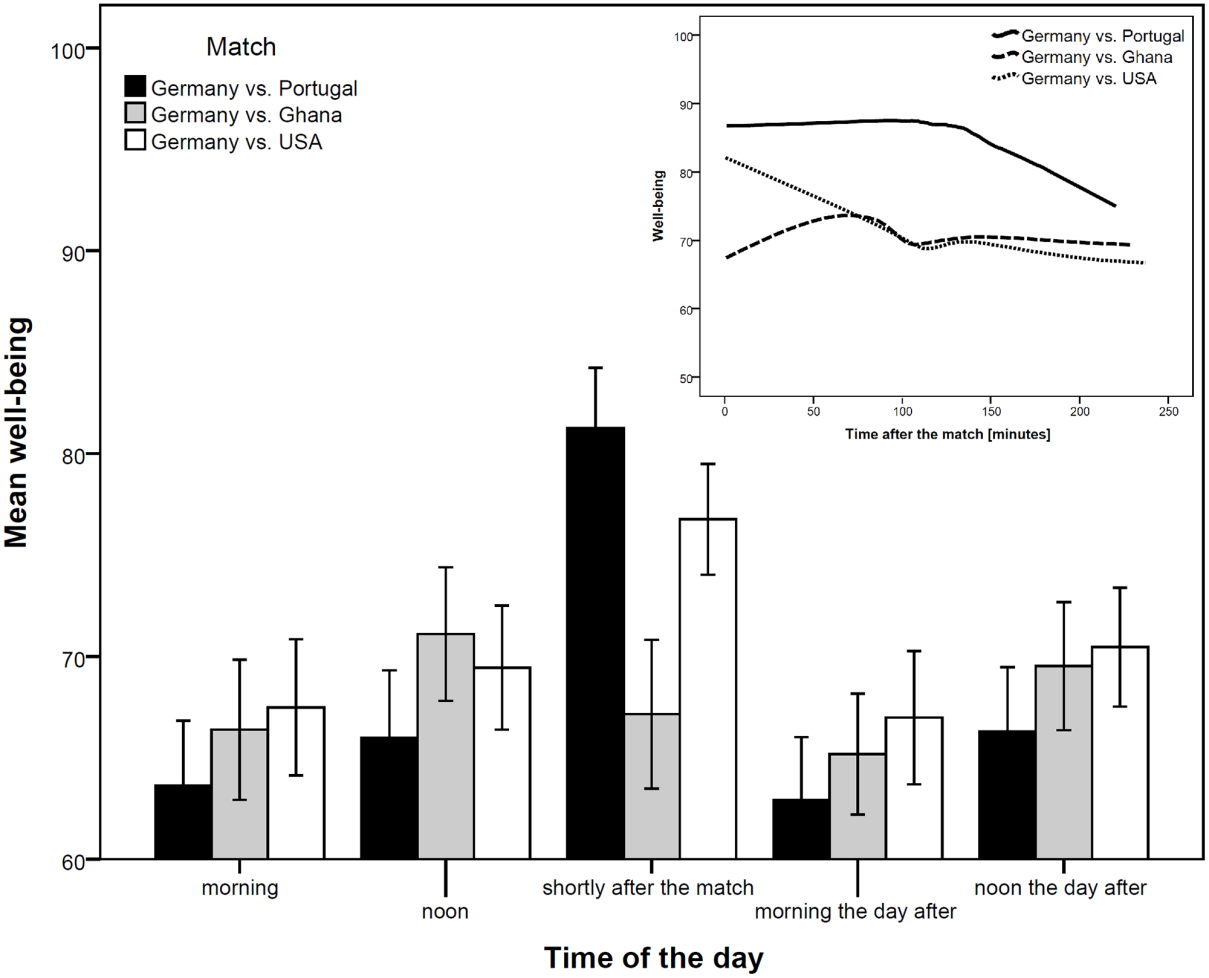
**Long and short-term influences of a soccer match win on subjective well-being.** Bar charts including 90% confidence intervals. Scatterplot with lines using Epanechnikov kernel density function (99%).

Third, we followed a within-subject design. One might argue that the design is quasi-experimental and there might be other differences between the soccer spectators and those who have not seen the matches which could account for the found differences in well-being. Furthermore, we were interested in how long a positive effect of won soccer matches on subjective well-being lasts. Therefore, we added to our analyses two time points before the match (i.e., morning and noon on the day of the respective match) and two time points after the match (i.e., morning and noon the day after the respective match). Repeated measures ANOVAs replicated the results of the between-subject view by finding significant interactions for the Portugal and USA match (*F* = 5.13, *p* < 0.001, $\eta_{p}^{2}$ = 0.048; and *F* = 2.49, *p* = 0.043, $\eta_{p}^{2}$ = 0.027, respectively) but no substantial effect for the Ghana match (*F* = 1.19, *p* = 0.314, $\eta_{p}^{2}$ = 0.015). Furthermore, as can be seen in **Figure 1**, the positive effect of a German win (against Portugal and USA) was already diminished by the next morning. Having a closer look by analyzing the exact time participants gave their feedback about their well-being after having received the reminder revealed that the positive effect on well-being due to a win lasted about 2 h for the match against Portugal and then started to fall (see scatter plot in the upper right corner of **Figure 1**; solid line in the chart). The effect for the win against the USA immediately started to fall (dotted line) and, as expected, no effect was found for the match against Ghana (dashed line)^2^.

## Discussion

The results of the current study showed that the well-being of soccer spectators directly measured after the soccer match was significantly and substantially higher compared with non-spectators, but only when the match was won by the German team. In the first match against Portugal, the German team scored four times and won the match. The rise in subjective well-being was substantial (Cohen’s *d* = 0.64). In the third match against the United States, the Germans won by one goal. Consequently, soccer spectators reported higher well-being than non-spectators (*d* = 0.49). Nevertheless, this effect was lower than the first. A possible explanation is that the impact’s power varies dependent on the strength of the opponent, expectations of the spectators, and/or the final goal difference.

According to this notion, Portugal was deemed a rather strong adversary, thus the fans were surprised by the overwhelming win. In contrast, when confronted with the US, participants’ expectations were likely to have been high because the team is not considered especially strong. Against this backdrop, it seems plausible that the fact that the Germans scored only one goal was disappointing despite the win, which might have weakened the observed effect on subjective well-being.

The second match against Ghana ended as a draw and the data showed no significant difference in well-being between soccer spectators and non-spectators (*d* = 0.06), although a mild trend toward lower spectators’ well-being became apparent. This trend points in the hypothesized direction, suggesting that a draw against an opponent, not expected to pose an actual challenge, as was the case for Ghana, would be associated with declines in well-being among soccer interested participants.

In sum, a win of a soccer match has a substantial positive effect on spectators’ subjective well-being. All the more, it seems reasonable to conclude that there are further determinants, like the strength of the opponent or the expectations of the viewers, which affect spectators’ subjective well-being. In order to gain a more elaborate impression of the involved mechanisms, future research should strive to identify and understand the nature of these factors, which is beyond the scope of the present article.

The second hypothesis stated that based on often described impressions, the effect on subjective well-being should be grounded in a general mechanism (e.g., emotional contagion) supporting well-being in every spectator not just those supporting the German team. The present study found no support for this assumption. Controlling for the strength of support of the German national soccer team caused all effects to vanish. Put differently, only active spectators who also supported the German soccer team showed a gain in subjective well-being, but not ordinary spectators. Furthermore, the effect shown by the spectators supporting the German team was short-lived, i.e., by the next day in the morning, the positive effect on subjective well-being was gone. This is clearly in opposition to often echoed assertions that national events (if won) have a positive effect on national sentiments (at least for subjective well-being; Maennig and Porsche, 2008; Pawlowski et al., 2014). Our results are in line with Elling et al. (2014) who found that international sport events led to small and short-termed changes in subjective well-being. However, it is important to mention that a positive effect might still be possible but only for more significant events such as winning the World Cup. Investigating such events might be a fruitful endeavor for future research.

### Future Research

As an array of new questions arises from the present insights, future research is required to refine the understanding of the involved dynamics and mechanisms as well as their interplay. To start with, previous research has emphasized that failures harbor the capacity to exert an even stronger impact on participants than successes. Given that our study was based on a natural intervention (i.e., the group stage of the World Cup), which is generally thought to be a useful way to conduct research without intervening to set up an experimental manipulation, the team’s matches, and their respective results could not be influenced. In consequence, we witnessed two wins and one draw. Hence, additional data for lost matches is needed to expand our knowledge on the link of subjective well-being and soccer match results.

Further, in looking for potential moderators and mediators to describe the observed relationship more accurately, it would be worthwhile to distinguish between fan types, supposing that they might deal with their team’s outcomes differently, which would in turn result in different patterns of subjective well-being following matches of their favorite team. Tackling this question, a taxonomy has been proposed that classifies spectators on two dimensions (hot vs. cold and traditional vs. consumer), to sort them into four categories (supporter, fan, follower, flâneur: Giulianotti, 2002). Similarly, Vallerand et al. (2008) applied the Dualistic Model of Passion to the soccer context, concluding that only harmonious passion, and not just any kind of strong identity involvement in team support is linked to psychological adjustment and positive outcomes (e.g., increased subjective well-being and self-esteem). Both models could be used to provide an orientation and guide further research in this direction.

Furthermore, it would be interesting to test whether the observed effects could be replicated at other mega events (e.g., the Olympics) and in different countries and cultures, such as the USA where soccer is not as deeply engrained in the philosophy of life and mentality, as in Germany. Finally, we recommend that future research examines the question of the longevity of the observed effects. So far this aspect remains rather puzzling: Traditional perspectives emphasize the fleeting nature of such impacts (Myers and Diener, 1995) which is in line with our research, as only the data collected directly after the matches showed significant effects, that had completely faded away the following morning. However, recently long-lasting health effects of euphoria, resulting from soccer matches have been found (Aboa-Eboulé et al., 2014), which call previous conceptualizations into question.

Finally, it might also make a difference whether soccer spectators or sporting event spectators in general watch the event alone or in a group. Being with excited people might enhance the effect of emotional contagion and, in turn, well-being. In other words, direct social interaction with spectators might elicit different effects than indirect social interactions, such as being alone and watching the excitement of spectators in the stadium.

### Limitations

Firstly, the group sizes were far from equal, with many more participants following the World Cup than disinterested non-spectators. This has slightly decreased the power to detect effects. Nevertheless, we were able to replicate the findings using a (more powerful) within-subject design by comparing several time points within each participant.

Secondly, we asked participants about their support in the German soccer team after the group phase and not before, i.e., it could be that the two wins of the German soccer team might have influenced participants’ support for the German team. Assessing the support at the beginning of the study might have revealed a more unbiased attitude toward the German soccer team. Although this is indeed a limitation, we think a possible effect was probably of rather low effect size. If we assume that a soccer win influences one’s support for the German soccer team, this effect should also raise the probability of seeing the next soccer match of the German team. As can be seen from **Table 1**, the number of spectators did not raise during the course of the group phase.

Thirdly, with regard to the applied methodology, the scientific community has been traditionally opposed single-item measures, claiming they lack the necessary psychometric properties to achieve accurate measurement, despite several studies having compiled evidence for the suitability of single-item measures in positive psychology (e.g., to assess self-esteem: Robins et al., 2001) and other fields of psychological research (Bergkvist and Rossiter, 2007). Similarly, self-reports have been criticized for the same reason, even though they yield psychometrically acceptable levels of validity and reliability when measuring subjective well-being (Diener, 1994).

Fourthly, a minor methodological drawback might also lie in the fact that participants were obliged to have a smartphone. Given that it has been shown that smartphone ownership and use are predicted by “Big Five” personality traits and demographics suggesting that extraverted, male, well educated, young participants are more likely to own a smartphone (Lane and Manner, 2011), one might argue that samples of smartphone studies suffer from shortcomings in terms of internal validity, representativeness, respectively. In a similar vein, it could be problematic that, due to technical incompatibilities, only owners of an Android smartphone were allowed to participate in our study, which could have paved the way for further biases. Nevertheless, according to recent statistics, about 80% of smartphones owners have an Android operating system^3^. Therefore, biases from different smartphone operating systems are probably minor.

## Conclusion

Despite these limitations, and even though it is clear that much work remains to be done, the present study adds to the scientific body of knowledge as, to our knowledge, it has been the first to illustrate the immediate effects of soccer results of the spectators’ supported team on subjective well-being. The outcomes of our analyses suggest a robust, medium – albeit short-lived – effect, which is based upon data that has been collected by means of a natural intervention (i.e., the soccer World Cup 2014), a method that is believed to yield highly externally valid data.

As for the applicability of science smartphone apps to run studies, our research has once more underscored its benefits (i.e., small costs, opportunity to collect vast data in the field, somewhat more diverse samples than college-based samples; for a discussion about smartphone apps in science, see Miller, 2012). Scholars are increasingly capitalizing on this pragmatic method to collect longitudinal data (e.g., Do and Gatica-Perez, 2014; Min et al., 2014). Such smartphone studies might even represent the future of some lines of research, as they enable scientists to collect a vast amount of ecologically valid data in an easy way. Furthermore, given that the market keeps growing, almost everyone will be in possession of a smartphone (Miller, 2012). Unlike previous research that was primarily carried out in laboratory settings, smartphone studies do not require a physical presence. This is seen as a considerable advantage, which provided us with the ideal methodological frame to examine immediate effects of soccer results on well-being in a geographically widely dispersed sample.

In a nutshell, we have extended the application of smartphone apps for scientific research purposes to the field of social and positive psychology, where it was successfully shown that the results of the soccer World Cup have the capacity to profoundly affect subjective well-being of large parts of the population.

## Author Contributions

SS was the principal investigator, conceived the study, contributed to the study design, data analyses, data management, writing of the manuscript, and programming of the Smartphone app. FMG contributed to the study design, data collection, and writing of the manuscript. FG contributed to the study design, data collection, and critically revised the manuscript. All authors have read and approved the final manuscript.

## Conflict of Interest Statement

The authors declare that the research was conducted in the absence of any commercial or financial relationships that could be construed as a potential conflict of interest.

## Footnotes

1 Effects of time and weekday were also calculated with ipsatized well-being scores (i.e., controlling for individual response tendencies by subtracting each well-being assessment from the participants mean well-being score). All conclusions remained the same (i.e., significant but very weak effects for the hour of the day and day of the week). Therefore, detailed results are omitted for brevity.

2 We also analyzed whether the number of matches watched could reveal additional conclusions. None of the variables under investigation (e.g., age, sex, mean well-being score, number of ratings) was significant. Therefore, detailed results are omitted for brevity.

3 http://en.wikipedia.org/wiki/Usage_share_of_operating_systems#Mobile_devices
